# Association of oncogenic bacteria with colorectal cancer in South China

**DOI:** 10.18632/oncotarget.13094

**Published:** 2016-11-04

**Authors:** Youlian Zhou, Hanchang He, Haoming Xu, Yingfei Li, Zhiming Li, Yanlei Du, Jie He, Yongjian Zhou, Hong Wang, Yuqiang Nie

**Affiliations:** ^1^ Department of Gastroenterology, Guangzhou Digestive Disease Center, Guangzhou First People's Hospital, Guangzhou Medical University, Guangzhou, 510180, China; ^2^ The First People's Foshan Hospital, Chancheng District, Foshan, 528000, Guangdong, China

**Keywords:** colorectal cancer, fusobacterium spp., enterococcus faecalis, enterotoxigenic bacteroidesfragilis, enteropathogenic escherichia coli

## Abstract

To quantify *Fusobacterium spp., Enterococcus faecalis (E.faecalis),* Enterotoxigenic *Bacteroides fragilis* (ETBF), and Enteropathogenic *Escherichia coli* in colorectal cancer (CRC) patients and their possible association with CRC clinicopathogical features, we collected the resected tumors and adjacent normal tissues (N) from 97 CRC patients. 48 age- and sex-matched healthy controls (HC) were also recruited. Real-time PCR was used for bacterial quantification. The median abundance of*Fusobacterium spp*.(*p* < 0.001, vs. *N*; *p* < 0.01,vs. HC), *E.faecalis* (*p* < 0.05, vs. *N*; *p* < 0.01, vs. HC) and ETBF (*p* < 0.001, vs. N; *p* < 0.05,vs. HC) in tumor tissues was significantly higher than that detected in normal tissue and HC. *E.faecalis* was detected in 95.88% of tumors and 93.81% of adjacent tissues. *Fusobacterium spp.* was detected in 72.16% of tumors and 67.01% of adjacent tissues. The combined *E.faecalis* and *Fusobacterium spp.* were detected in 70.10% of tumors and 36.08% of adjacent normal tissues. All four bacteria were detected in 33.72% and 22.09% of paired tumor and adjacent normal tissues, respectively. *E.faecalis* and *Fusobacterium spp.* are enriched in both tumor and adjacent tissue of CRC patients when compared to HC, suggesting that it is possible to be previously undetected changes in the pathohistologically normal colon tissue in the proximity of the tumor.

## INTRODUCTION

Many studies have established a causal link between bacterial and viral infections and cancers like for instance the link between Human papilloma virus and cervical cancer [1], *H.pylori* and gastric carcinoma [2], Hepatitis B and C virus and hepatocellular carcinoma [3, 4]. Colorectal cancer is one of the most common types of cancer and the third cause of cancer mortality worldwide [5]. Its etiology is still not fully understood. The possible influence of oncogenic bacteria in its development was first suggested in a case report from 1950s when a clinical association between *Streptococcus bovis* bacteraemia/endocarditis and carcinoma of the sigmoid was reported [6]. Since then, efforts have been made to explore the possible pathogens involved in CRC development. Multiple studies have demonstrated enrichment of fecal or tissue samples of CRC patients with specific bacterial pathogens, including *Fusobacterium spp*. [7–9], *Enterococcus faecalis* (*E.faecalis*), [10], Enterotoxigenic *Bacteroides fragilis* (ETBF), Enteropathogenic *Escherichia coli* (EPEC) [11] and *Streptococcus gallolyticus (S.gallolyticus*) [12]. Recently, *Nakatsu et al.* suggested a taxonomically defined microbial consortium implicated in the development of CRC [13]. In addition, *Ericsson et al.* identified a naturally occurring variation in gut microbes associated with severity of colorectal cancer, as well as the abundance of certain taxa associated with decreased tumor burden [14]. Indeed, it seems that once the key players in the microbial dysbiosis associated with colorectal carcinogenesis are identified, this would probably result in new strategies in the diagnosis, treatment and even prevention of CRC.

Based on both *in vitro* and *in vivo* studies, oncogenic mechanisms of bacteria-driven CRC tumorigenesis include Wnt signaling activation (ETBF [15], EPEC [11], *Fusobacterium* [16]), pro-inflammatory signaling (*E. faecalis* [17, 18], *S. gallolyticu* [19, 20]) and genotoxicity (EPEC [21], AIEC [22–24]).

To date, however, the presence of multiple CRC-associated bacteria have not been identified in populations of South China. And these types of studies were mostly performed in Western countries where the genetic and ethnic backgrounds of patients differ from those in Asian regions, which in turn may affect the composition of gut microbiota [25, 26]. Furthermore, in these studies, pathogens, reported to be associated with CRC [27], have only been quantified in paired adenocarcinoma and adjacent normal mucosal samples, or fecal samples of CRC patients, without the comparison with the age- and sex-matched healthy control population. As samples in these studies were collected from patients already diagnosed with CRC, additional changes of gut microbiota might have occured. Those patients might be more susceptible to microflora colonization of normal colon epithelium––not only in existing cancer tissue, but also in the macroscopically normal adjacent tissue, which may indicate a pre-existing risk to bacteria colonization/infection. For instance, 16SrDNA profile of colorectal cancer (CRC) paired tumor and normal biopsies has suggested that only 3% of biopsies from healthy controls contained any type of bacteria, while ~90% patients with carcinomas or adenomas had 10^3^–10^5^microbiota in both macroscopically normal and malignant tissues [28].

In the present study, we applied quantitative real-time polymerase chain reaction (qPCR) to detect the presence of four pathogens (*Fusobacterium spp., E.faecalis,* ETBF and EPEC) in paired adenocarcinoma and adjacent normal mucosal samples, as well as in age- and sex-matched healthy controls in population of South China.

## RESULTS

### Clinicopathological characterization of CRC patients

The clinicopathological characteristics of the patients are summarized in Table 1. In brief, a total of 61 men and 36 women with a median age of 64.58 years (range, 31–92 years) were included in the study. The majority of cases were stage II or III cancers (64.94%), while stage I and IV cancers accounted for 9.28% and 25.17%, respectively. The cohort consisted of 64.95% rectal and 35.05% colon cancers, with moderately or well differentiated tumors accounting for 91.75% of analyzed samples.

**Table 1:** Clinicopathological characteristics of CRC patients

	CRC patients (*n* = 97)
Male/Female (n (%))	61 (62.89%)/36(37.11%)
Age (Mean ± SE)	64.5773 ± 1.1973
Tumor location (n (%))	
Colon cancer	63 (64.95%)
Rectal cancer	34 (35.05%)
Differentiation (n (%))	
Low differentiation	8 (8.25%)
Moderate-well differentiation	89 (91.75%)
TNM staging (n (%))	
T1 + T2	16 (16.49%)
T3 + T4	81 (83.51%)
Lymphatic metastasis (n (%))	
N0	57 (58.76%)
N1 + N2	40 (41.24%)
Metastasis (n (%))	
M0	74 (76.29%)
M1	23 (23.71%)
Dukes' classification (n (%))	
A	9 (9.28%)
B	26 (26.80%)
C	37 (38.14%)
D	25 (25.78%)

### *Fusobacterium spp., E.faecalis* and Enterotoxigenic *Bacteroides fragilis* (ETBF) are significantly enriched in CRC tissues compared to the adjacent normal colon tissue

**Figure 1:**
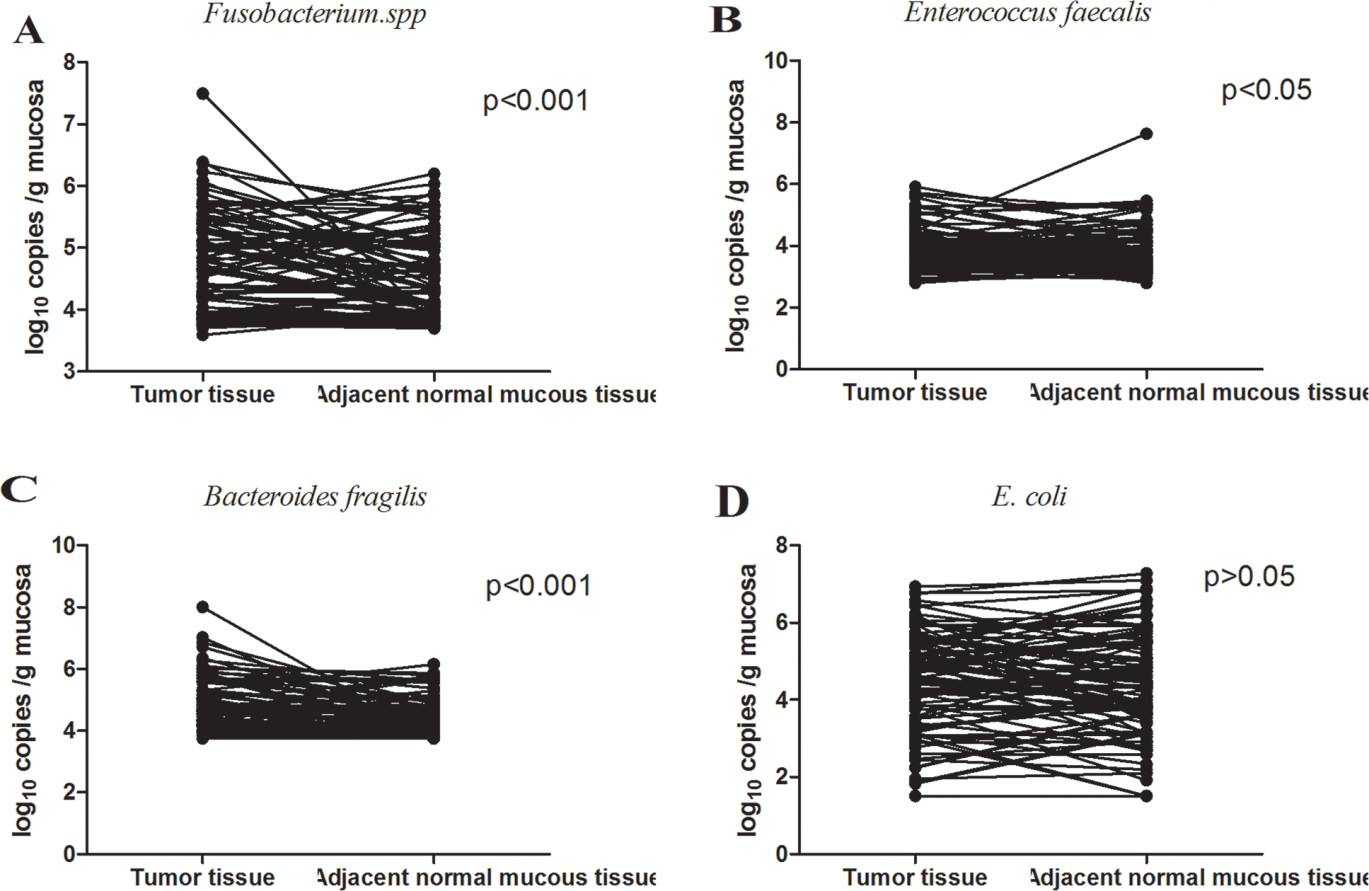
Quantitative real-time PCR analysis of bacteria in CRC tumor and corresponding normal mucous samples, presented as log10 copies/g mucosa of 50 ng DNA The median abundance of *Fusobacterium spp.* (*N* = 97, *p* < 0.001, Wilcoxon signed rank test), *E.faecalis* (*N* = 97, *p* < 0.05, Wilcoxon signed rank test) and ETBF (*N* = 87, *p* < 0.001, Wilcoxon signed rank test) in CRC tissues was significantly higher than that in adjacent normal mucous tissues (10 cm beyond cancer margins), while there was no significance in E.coli (*N* = 96, *p* > 0.05, Wilcoxon signed rank test) between CRC tissue and adjacent normal mucous tissue.

In our study, CRC-associated bacteria were quantified in adenocarcinoma and adjacent normal mucous samples of the same patient by qPCR, using a serial dilution of genomic DNA of each bacterium as standard. As a result, varying levels of bacterial colonization in tumor and/or adjacent normal mucosa for all bacteria were detected. Of the bacteria that were examined, the median abundance of *Fusobacterium spp.* (*p* < 0.001, Wilcoxon signed rank test), *E.faecalis* (*p* < 0.05, Wilcoxon signed rank test) and ETBF (*p* < 0.001, Wilcoxon signed rank test) in CRC tissues was significantly higher than that in corresponding adjacent normal mucous tissue (10 cm beyond cancer margins). The results of these analyses are summarized in Figure 1.

### *Fusobacterium spp.* and *E.faecalis* are increased in the adjacent normal mucous tissue of CRC patients compared to the healthy controls

**Figure 2:**
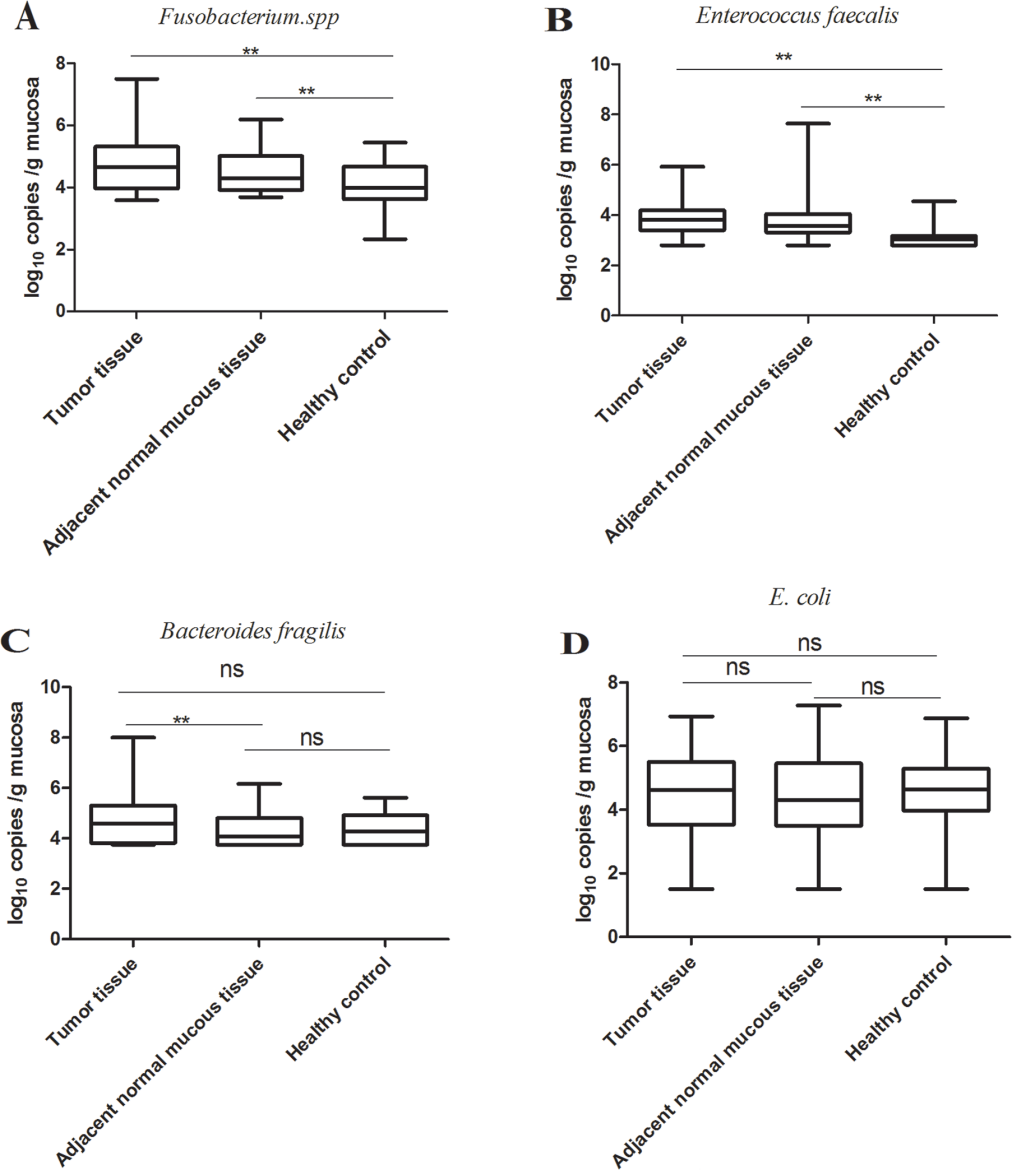
Quantification of bacteria in tumor tissues, adjacent normal mucous tissues of CRC patients and control tissues from age- and gender-matched healthy volunteers *Fusobacterium spp.* (N_CRC_ = 97, N_HC_ = 48; *p* < 0.05, Kruskal-Wallis test followed by multiple comparisons) and E.faecalis (N_CRC_ =97, N_HC_ =48; *p* < 0.05, Kruskal-Wallis test followed by multiple comparisons) were markedly enriched in the matched adjacent normal mucous tissues compared to the healthy controls. No difference in *E.coli* (N_CRC_ = 96, N_HC_ = 48; *p* > 0.05) and *Bacteroides fragilis* (N_CRC_ =87, N_HC_ =48; p > 0.05) was observed between tumor tissues, corresponding adjacent normal mucous tissues and healthy controls. **p < 0.05; ns, no significance. N_CRC_, the number of CRC patients; N_HC_, the number of healthy controls.

To determine whether CRC patients may be susceptible to CRC-associated bacteria colonization of the normally sterile colonic epithelium––the surrounding macroscopically normal tissue, we have examined the bacterial status of tumor tissue and adjacent normal mucous tissues of CRC patients as well as the bacterial status of age- and sex-matched healthy controls. *Fusobacterium spp.* (*p* < 0.05, Kruskal-Wallis test followed by multiple comparisons) and *E.faecalis* (*p* < 0.05, Kruskal-Wallis test followed by multiple comparisons) were markedly enriched in the adjacent normal mucous tissue of CRC patients compared to healthy controls (Figure 2). No difference however was observed for EPEC (*p* > 0.05) between tumor tissues, adjacent normal tissues of CRC patients and healthy controls. *E.faecalis* was the most common bacteria detected, occurring in 95.88% (*N* = 93) of tumors and 93.81% (*N* = 91) of adjacent normal mucous tissues. The second most common bacteria detected in our study was *Fusobacterium spp.* which was detected in 72.16% (*N* = 70) of tumors and 67.01%(*N* = 65) of adjacent normal mucous tissues (Table 3). Combined *E.faecalis* and *Fusobacterium spp.* were detected in70.10%(*N* = 68) of tumor samples and 36.08% (*N* = 35) of adjacent normal mucous tissues. The combination of all examined bacterial species was detected in 33.72% (*N* = 29) of tumor tissues and 22.09%(*N* = 19) of adjacent normal mucous tissues (Table 2).

**Table 2:** Gut microbiota in tumor and adjacent normal tissue of CRC patients

	Tumor tissue	Adjacent normal tissue
*Fusobacterium spp.*	70 (72.16%)**	65 (67.01%)**
*B.fragilis*	56 (64.37%)	40 (45.98%)
*E.faecalis*	93 (95.88%)**	91 (93.81%)**
*E. coli*	47 (48.96%)	40 (41.67%)
*Fusobacterium spp. + B.fragilis*	49 (56.32%)	32 (36.78%)
*Fusobacterium spp. + E.faecalis*	68 (70.10%)**	35 (36.08%)
*Fusobacterium spp. + E.coli*	36 (37.50%)	26 (27.08%)
*B.fragilis + E.faecalis*	55 (63.22%)	37 (42.53%)
*B.fragilis + E.coli*	35 (40.70%)	24 (27.91%)
*E.faecalis + E.coli*	46 (47.92%)	39 (40.63%)
*Fusobacterium spp. + B.fragilis + E.faecalis*	47 (54.02%)	29 (33.33%)
*Fusobacterium spp. + B.fragilis + E.coli*	30 (34.88%)	20 (23.26%)
*B.fragilis + E.faecalis + E.coli*	34 (39.53%)	23 (26.74%)
*Fusobacterium spp. + B.fragilis +*	29 (33.72%)	19 (22.09%)
*E.faecalis + E.coli*		

** high abundance in CRC.

**Table 3:** Correlation between bacterial population and clinicopathological parameters

Spearman's rho	*Fusobacteriumspp.* (r, p)		*B.fragilis* (r, p)		*E.faecalis* (r, p)		*E.coli* (r, p)	
Tumor location	−0.12	0.243	−0.166	0.117	−0.062	0.546	−0.256	0.012
Tumor differitation	0.078	0.452	0.073	0.496	0.041	0.69	0.006	0.957
Tumor infiltration	−0.06	0.561	−0.108	0.302	−0.163	0.144	−0.015	0.887
Lymphatic metastasis	−0.02	0.847	−0.03	0.778	−0.19	0.064	−0.14	0.177
metastasis	0.049	0.631	0.04	0.708	−0.233	0.022	−0.219	0.032
Duke's stage	−0.108	0.294	−0.078	0.468	−0.192	0.061	−0.145	0.162
Tissue CEA	−0.252	0.043	−0.162	0.216	0.028	0.825	0.078	0.542
Tissue P53	−0.156	0.15	−0.075	0.512	0.074	0.500	0.017	0.879
Tissue Villin	−0.073	0.522	−0.121	0.307	0.047	0.68	0.046	0.685
Tissue EGFR	−0.107	0.415	−0.136	0.322	−0.005	0.967	0.163	0.212
Tissue Ki67	−0.037	0.73	−0.113	0.309	0.071	0.505	0.067	0.535
Serum CEA	0.109	0.303	0.194	0.076	0.112	0.286	−0.049	0.642
Serum CA199	0.013	0.900	0.000	0.999	−0.018	0.868	−0.204	0.550
Serum CRP	0.03	0.786	0.09	0.434	0.261	0.016	−0.082	0.461
*B.fragilis*	0.631	0.000**			0.2	0.054	0.347	0.01**
*E.faecalis*	0.067	0.508	0.02	0.54			0.257	0.01**
*E. coli*	0.199	0.048	0.347	0.001**	0.257	0.010**		

** *p* < 0.05.

### Clinicopathological features of CRC patients and their bacterial status

Clinicopathological features of CRC patients and their association with the examined bacterial status are summarized in Table 3. No strongly significant association of four examined bacteria iwith CRC clinicopathologica lparameters e.g. tumor stage, location, infiltration depth and pathological differentiation were observed (*p* > 0.05). On the other hand an association between *Fusobacterium spp. and B.faecalis* in CRC samples was observed (*r* = 0.631, *p* = 0.000)(Table 3).

## DISCUSSION

It is known that bacterial dysbiosis contributes to a variety of colorectal diseases including CRC, but no specific bacterium was confirmed to be the key virulence factor [29–32]. Although the bacterial status of CRC patients previously has been explored before [27], the data on the CRC-associated microbes in Chinese CRC populations are scarce. Therefore, in order to gain a better understanding of bacterial colonization patterns in Chinese CRC patients in this study we have examined the presence of four CRC-associated bacteria across a single Chinese cohort in both tumor and histologically normal adjacent mucous tissue of CRC patients, as well as in age- and sex-matched healthy controls.

Notably, our finding that *Fusobacterium spp.*, *E.faecalis* and Enterotoxigenic *Bacteroides fragilis* (ETBF) are significantly enriched in CRC tissues compared to the adjacent normal mucous tissues is consistent with previous studies [8, 27, 33]. *Viljoen et al.* [27] found a positive association between high-level colonization by *Fusobacterium* and regional lymph node metastases. In addition, in the same study the ETBF colonization and high-level of *Fusobacterium* colonization were associated with late-stage CRC, which we however did not observe in our present study. Colorectal cancer tissues provide rich nutrition surface and tumor-homing activity of certain microbiota has been reported in the literature [34]. Nevertheless, the microbiota presence does not necessarily suggest their oncogenic potential. Therefore, evaluating distribution of microflora in relation to lifestyle, ethnicity and clinicopathological factors may be essential in assessing the host-susceptibility to infection and putative bacteria-associated oncogenic mechanisms. In addition, microbiota abundance is not the only parameter that may be correlated with clinicopathological features of patients since even low-abundant microflora may exert significant effect on the host through toxins secretion. For instance, *Dutilh et al.* suggested that enterobacterial toxins were among the most highly detected in the meta transcriptomic sequencing data of CRC tumors and matched adjacent normal samples [35]. Indeed, antigens and metabolites produced by gut microbiota may have important roles in affecting CRC risk through their interactions with host immunity andmetabolism [36, 37]. The observed changes in bacterial mocrobiome might contribute to the further progression of CRC through the various possible mechanisms. In addition, it is possible that changes of gut microbiota identified in the present study might be a consequence of CRC.

In our study a group consisting of healthy controls was included in order to obtain punch biopsies of normal mucosal tissues and to compare them with the normal mucous tissues in the proximity of the tumors of CRC patients. In our study, *Fusobacterium spp.* and *E.faecalis* were increased in the adjacent normal mucous tissues of CRC patients compared to the healthy controls. These findings may be an additional supporting evidence for the hypothesis of the possible association of *Fusobacterium spp.* and *E.faecalis* with the transformation of colorectal mucosa from early adenomatous polyp stages to late CRC stages. In fact, it is possible that increase in *Fusobacterium spp.* and *E.faecalis* might be an earlier event than adenoma. However, this hypothesis needs a prospective study to determine the possible association of mentioned benign colorectal lesions with the increase in *Fusobacterium spp.* and *E.faecalis* presence.

There are some limitations to our study. First, due to the study design it is impossible to untangle the causal relationship between gut microflora and CRC. As samples were collected from patients already diagnosed with CRC, whether *Fusobacterium spp.* or *E.faecalis*is a cause or a consequence of CRC is not clear. Second, the sample size included in this study was rather small. In addition, clinicopathological features such as stage of disease [27], tumor location [27], age [27], lymph node metastases [7, 38] which have been previously reported to be associated with high-level colonization by *Fusobacterium* in patients with CRC were not observed in our study. Due to the lack of data on follow-up, we could not evaluate these bacteria in association with a longer time-to-relapse. In this study, we have examined the presence of four pathogens in tissue samples of CRC patients not in fecal samples. Indeed, adherent microflora might have a greater influence on gene expression in the colonic mucosal cells than transient microbiota that are flushed in faecal samples. Nevertheless. Larger studies of faecal and colonic tissue samples from different stages of CRC are necessary to determine possible bacterial biomarkers of oncogenic transformation.

## MATERIALS AND METHODS

### Patients

In this study, 97 patients with pathologically confirmed colorectal adenocarcinoma who underwent the surgical resection at the Department of General Surgery of Guangzhou First People's hospital between November 2012 and November 2014 were recruited. The medical history of recruited patients was evaluated and no one had history of gastrointestinal disease, ulceration or other disease that might affect gut microbiota. Dietary preferences and antibiotic usage (in the last four weeks) of enrolled subjects were recorded to role out diet habit bias or antibiotic usage that may influence the bacterial microbiome. Fresh CRC and adjacent non-tumor tissues (10 cm beyond cancer margins) from each subject were collected. In addition, 48 age- and sex- matched control subjects were recruited. They were referred to hospital for colonoscopy for various reasons and no gastrointestinal disease or history of gastrointestinal diseases and ulcerations were reported and normal colonic mucosa was confirmed. Colonoscopy biopsy was taken from control subjects to compare their level of microbes with those in CRC tissues and adjacent normal tissues taken from the same patient.

Samples were snap frozen in liquid nitrogen and then stored at −80°C until use. The CRC stages were assigned according to TNM and Dukes grades [39]. All participants were local residents of Guangzhou city for more than 10 years.

All study protocols were in compliance with the Declaration of Helsinki and were approved by the Ethics Committee of Guangzhou First People's Hospital affiliated with Guangzhou Medical University. Informed consent was obtained from all subjects. All experiments were performed in accordance with relevant guidelines and regulations.

### DNA isolation and quantitative real- time PCR (qPCR)

Total DNA was isolated from samples using QIAamp DNA Mini Kit (QIAGEN, Hilden, Germany) according to the manufacturer's instructions. All DNA samples were stored at –80°C until further processing.

Primer pairs targeted to detect 16S ribosomal RNA gene of four bacterial groups or species included in this study (*Fusobacterium spp*., *E.faecalis*, ETBF and EPEC) are listed in Table 4. The 16S rRNA gene of each bacterial strain was cloned into the pUCm-T vector (Sangon, Shanghai, China) according to the manufacturer's protocol and was used as a copy number standard. For each real-time PCR standard, copy number concentration was calculated, based on the length of the PCR product and the mass concentration. The standards were stored at –80°C, and serial dilutions (1 to 10^8^ copies/μL) were made prior to each real-time PCR assay. The results of qPCR for each sample were expressed as the copy number of bacterial 16S ribosomal DNA per gram of tissue. Real-time PCR assays were performed in 96-well optical plates using the LightCycler^®^ 480 Real-Time PCR System (Roche Diagnostics, Rotkreuz, Switzerland). All assays were carried out in duplicate and performed in a total volume of 20μl with LightCycler^®^ 480 SYBR Green I Master solution (Roche Diagnostics, Mannheim, Germany). The reaction mixture consisted of 0.5 μM of each of the specific primer pairs and 5 μl of DNA template. Amplifications were performed as follows: initial denaturation at 95°C for 5 min, followed by 45 cycles of denaturation at 95°C for 10 s, annealing at 52–56°C (primer dependent) for 10 s, and extension at 72°C for 10 s. Specificity of each amplification was assessed by melting curve analysis of the amplified PCR product. The efficiency of amplification for each primer pair was estimated from the standard curves.

**Table 4:** Primers for the detection of specific bacterial pathogens by quantitative real-time PCR

Target bacteria	Primer Sequence (5′ to 3′)	Annealing Temp (°C)	Product Size (bp)
*Fusobacterium spp.* [40]	F: GGATTTATTGGGCGTAAAGC	55	162
	R:GGCATTCCTACAAATATCTACGAA		
*Enterococcus faecalis* [41]	F: CCCTTATTGTTAGTTGCCATCATT	55	144
	R: ACTCGTTGTACTTCCCATTGT		
*B. fragilis* [42]	F: ATAGCCTTTCGAAAGRAAGAT	52	501
	R: CCAGTATCAACTGCAATTTTA		
*E. coli* [43]	F:GTTAATACCTTTGCTCATTGA	56	340
	R:ACCAGGGTATCTAATCCTGTT		

### Assessment of clinical parameters

Clinical parameters of CRC patients included in this study were collected according to medical history and medical records of hospital patients. The collected data included patients' gender, age, serological examinations such as serum CEA, CA199, C-reaction protein (CRP), and pathohistological biopsy immunohistochemical results for tissue CEA, P53, Villin, Epidermal Growth Factor Receptor (EGFR) and Ki67.

### Statistical analysis

All data are presented as means ± standard error mean (SEM) for quantitative variables and as frequencies for qualitative variables. Given the non-normal distribution of the data analyzed, the nonparametric test was used to examine changes in bacterial number. In order to assess quantitative differences between paired tumor and adjacent non-tumor samples for each bacterium, we used the Wilcoxon signed rank test and applied it to the subset of samples. For the comparison among healthy control, adjacent tissue and cancer tissue groups, Kruskal-Wallis test was applied. The Spearman correlation coefficient was calculated to estimate the linear correlations between variables. Statistical analyses were performed with the statistical software package SPSS16.0 (SPSS Inc., Chicago, IL, USA). Two-tailed *P value* less than 0.05 was considered statistically significant.
